# Rubidium Chloride Targets Jnk/p38-Mediated NF-κB Activation to Attenuate Osteoclastogenesis and Facilitate Osteoblastogenesis

**DOI:** 10.3389/fphar.2019.00584

**Published:** 2019-05-22

**Authors:** Zhengxiao Ouyang, Qianli Huang, Bin Liu, Hong Wu, Tang Liu, Yong Liu

**Affiliations:** ^1^State Key Laboratory of Powder Metallurgy, Central South University, Changsha, China; ^2^Department of Orthopedics, The Second Xiangya Hospital, Central South University, Changsha, China

**Keywords:** rubidium chloride, osteoclast, osteoblast, MAPK, NF-κB, osteoporosis

## Abstract

The unbalanced crosstalk between osteoclasts and osteoblasts could lead to disruptive bone homeostasis. Herein, we investigated the therapeutic effects of rubidium chloride (RbCl) on ovariectomized (OVX) and titanium (Ti) particle-induced calvaria osteolysis mouse models, showing that non-toxic RbCl attenuated RANKL-stimulated osteoclast formation and functionality while significantly enhancing osteogenesis *in vitro*. The expressions of osteoclast-specific genes were downregulated considerably by RbCl. Despite the direct inhibition of RANKL-induced activation of MAPK signaling, RbCl was able to target NF-κB directly and indirectly. We found that after the co-stimulation of the c-Jun N-terminal kinase (Jnk)/p38 activator and RANKL, RbCl inhibited the elevated expression of p-IKKα and the degradation of IκBα in osteoclast precursors, indicating indirect NF-κB inhibition *via* MAPK suppression. Furthermore, the two animal models demonstrated that RbCl attenuated tartrate-resistant acid phosphate (TRAP)-positive osteoclastogenesis and rescued bone loss caused by the hormonal dysfunction and wear particle *in vivo*. Altogether, these findings suggest that RbCl can target Jnk/p38-mediated NF-κB activation to attenuate osteoclastogenesis, while facilitating osteoblastogenesis both *in vivo* and *in vitro*, suggesting the possible future use of RbCl for surface coating of orthopedic implant biomaterials to protect against osteoporosis.

## Introduction

Bone is a dynamically balanced tissue that is consistently regulated by the crosstalk between bone resorption by osteoclasts and bone formation by osteoblasts (Qiao and Tang, 2018; Rao et al., 2018). The concord of osteoclast and osteoblast maintains bone homeostasis *via* vivid bone remodeling, contributing to the normal physiology of the skeletal system (Seeman and Delmas, 2006). By contrast, the disruption of this equilibrium inevitably results in a series of bone disorders, leading to afflicted skeletal physiology and functionality (Qu et al., 2018). The dysregulated maturation of osteoclasts and inhibited activation of osteoblasts show a considerable morbidity with compromised bone strength and increased bone fragility (Chen et al., 2017; Jing et al., 2018); the condition requires an increasing number of therapeutic remedies. Additionally, it is of interest to uncover novel strategies that combine effects of anti-osteoclastogenesis and pro-osteoblastogenesis simultaneously, which would definitely contribute to the development of an effective treatment against osteoporosis.

Importantly, the mitogen-activated protein kinase (MAPK) pathway that comprises the c-Jun N-terminal kinase (Jnk), extracellular-signal-regulated kinase (Erk), and p38 has been considered to promote osteoclastogenesis (Liu et al., 2017; Qiao et al., 2017). Likewise, nuclear factor κB (NF-κB) signaling has also been proven to play a crucial role during osteoclast formation (Ouyang et al., 2014; Qiao et al., 2016). Various reports showed the underlying interaction between MAPK and NF-κB (Dhawan and Richmond, 2002; Papa et al., 2004). By activating the p38/MAPK molecule, NF-κB appears to emerge *via* a Cdc42-independent mechanism (Korus et al., 2002). The accumulation of Jnk expression leads to the activation of NF-κB during macroautophagy, resulting in a rescued cancer cell survival (Yang et al., 2017). However, despite numerous studies on osteoporosis that highlight the roles of MAPK and NF-κB during osteoclastogenesis, few of them focus on the connection between MAPK and NF-κB. Therefore, targeting MAPK-mediated NF-κB activation may serve as a novel strategy in treating osteoporosis with an effective cascade-controlling response.

Considering the crucial roles of MAPK-mediated NF-κB activation during osteoclastogenesis, we seek to find novel compounds that could inhibit both MAPK and NF-κB activation, thereby effectively attenuating osteoclast formation. Herein, rubidium chloride (RbCl) that normally acts as a perfusion biomarker (Hougardy et al., 2003) and an anti-depressant (Kordjazy et al., 2015) was found to target Jnk/p38 in osteoclast precursors to inhibit osteoclast formation. More importantly, despite significant anti-osteoclast effects, RbCl could simultaneously promote osteoblastogenesis, thereby promising to satisfactorily treat osteoporosis. Also, few reports have covered the regulatory effects of RbCl in bone homeostasis. Therefore, we employed RbCl to provide therapeutic benefits for osteoclast and osteoblast, aiming at re-establishing the homeostasis of the bone microenvironment and elucidating the underlying molecular mechanisms, thus suggesting potential clinical uses of RbCl, such as in surface coating of orthopedic implants.

## Materials and Methods

### Main Reagents and Cell Culture

Recombinant macrophage colony stimulating factor (MCSF) and receptor activator for the nuclear factor-κB ligand (RANKL) were purchased from R&D Systems (Minneapolis, MN, USA). RbCl and fetal bovine serum (FBS) were purchased from Sigma-Aldrich (St. Louis, MO, USA). MAPK activator anisomycin and Asiatic acid were purchased from Sigma-Aldrich. Antibodies for western blotting and immunohistochemical analyses were purchased from Cell Signaling Technology (Cambridge, MA, USA).

Osteoclast precursor RAW 264.7 cells and osteoblast MC3T3-E1 cells were cultured in standard α-MEM medium supplemented with 10% FBS and 1% penicillin/streptomycin. Primary murine bone marrow monocytes (BMMs) were harvested from the femurs and tibiae of four- to six-week-old C57BL/6 mice and incubated in α-MEM medium with 30 ng/ml MCSF and 10% FBS. All cells were kept in a sterile condition at 5% CO_2_ of 37°C.

### Cell Viability

The viability of BMMs was assessed with the Cell Counting Kit-8 (CCK-8) method (Luo et al., 2018; Ouyang et al., 2018, 2019a,b; Xiao et al., 2018; Zhang et al., 2018). Briefly, BMMs were first seeded into 96-well plates at a density of 1.0 × 10^5^ cells/well for 24 h. Next, BMMs were treated with corresponding concentrations of RbCl (0, 0.1, 0.2, 0.4, 0.8, 1.6, 3.2, 6.4, 12.8, and 25.6 mM) for up to 4 days. A 10 μl CCK-8 solution mixed in 100 μl non-serum α-MEM medium was added into plates in 5% CO_2_ 37°C for 2 h. Optical density at 450 nm (OD_450_) was measured.

### Osteoclastogenesis *in vitro*

BMMs and RAW 264.7 cells seeded in 96-well plates were treated with RbCl at different concentrations (0.8, 1.6, and 3.2 mM). Cell medium with 50 ng/ml RANKL was changed every other day continuously until the 7th day. After osteoclast maturation, cells were fixed in 4% paraformaldehyde (PFA) and then rinsed with PBS. Tartrate-resistant acid phosphatase (TRAP) staining was then deployed to indicate the differentiated multi-nucleated osteoclasts. Stained osteoclasts with at least three nuclei were classified as TRAP-positive osteoclasts (Zhai et al., 2014).

### F-Actin Ring Immunofluorescence

F-actin ring was observed to indicate the ruffled membrane of osteoclasts (Xiao et al., 2015). Differentiated BMMs treated with RANKL (50 ng/ml) and RbCl (0.8, 1.6, and 3.2 mM) were fixed in 4% PFA and permeabilized in 0.1% Triton X-100. Alexa Fluor 647 phalloidin was then used to stain the osteoclast cytoskeleton. DAPI indicated cell nuclei. After several rinses with PBS, the fluorescent F-actin ring was observed under confocal microscopy (Leica TCS SP8, Germany).

### Bone Resorption Pit Evaluation

BMMs were seeded onto bovine bone slices at a density of 7,000 cells/slice in complete α-MEM medium with RbCl (0.8, 1.6, and 3.2 mM), RANKL (50 ng/ml), and MCSF (50 ng/ml). After the formation of matured osteoclasts, the adherent cells were removed by mechanical agitation and sonication. Scanning electron microscopy (SEM) was then used to visualize the osteolytic resorption pit.

### Quantitative Real-Time PCR

After treatment with RbCl (3.2 mM) and RANKL for 7 days, the BMMs seeded in 6-well plates were harvested to extract total RNA using RNeasy Mini Kit (Qiagen, CA, USA) following the manufacturer’s instructions. cDNA was synthesized from extracted RNA with reverse transcriptase kit (Takara Biotechnology, Japan). Real-time PCR was performed with SYBR Premix Ex Taq Kit (Takara Biotechnology, Japan) using ABI 7500 Sequencing Detection System (Applied Biosystems, Foster City, CA, USA), according to protocol under delta-delta-CT method. Mouse primers for CTR, CtsK, NFATc-1, TRAP, and GAPDH were listed as follows: CTR forward 5′-TGCAGACAACTCTTGGTTGG-3′ and reverse 5′-TCGGTTTCTTCTCCTCTGGA-3′; CtsK forward 5′-CTTCCAATACGTGCAGCAGA-3′ and reverse 5′-TCTTCAGGGCTTTCTCGTTC-3′; NFATc-1 forward 5′-CCGTTGCTTCCAGAAAATAACA-3′ and reverse 5′-TGTGGGATGTGAACTCGGAA-3′; TRAP forward 5′-CTGGAGTGCACGATGC-CAGCGACA-3′ and reverse 5′ -TCCGTGCTCGGCGATGGACCAGA-3′; GAPDH forward 5′-ACCCAGAAGACTGTGGATGG-3′ and reverse 5′-CACATTGGGGGTAGGAACAC-3′.

### Western Blotting

RAW 264.7 cells seeded in 6-well plates were pre-treated with or without 12.8 mM RbCl for 4 h. Afterward, 50 ng/ml RANKL was administered at specific time points (0, 5, 10, 20, 30, and 60 min) to determine the RANKL-activated expression of MAPK and NF-κB. Total protein was harvested with radioimmunoprecipitation assay (RIPA) lysis buffer (Beyotime Bioscience, Shanghai, China) supplemented with 1 mM phenylmethylsulfonyl fluoride (PMSF) and quantified with a BCA Protein Assay Kit (Thermo Scientific, IL, USA). Lysates were diluted in a loading buffer and denatured at 99°C for 10 min. Thirty micrograms of lysate were first resolved in sodium dodecyl sulfate-polyacrylamide gel electrophoresis (SDS-PAGE) on 10% precast gel at 80 V for 30 min and 120 V for 1 h, and then transferred to activated polyvinylidene fluoride (PVDF) membrane for 2.5 h at 250 mA. Five percent non-fat milk powder dissolved in Tris-buffer saline containing 0.05% Tween (TBST) was used to block membrane, and the first antibody was administered overnight at 4°C. After several rinses with TBST, the membrane was probed with corresponding secondary antibody for 1 h, followed by the visualization of protein bands using the Odyssey Infrared Imaging System (LI-COR Bioscience, NE, USA).

### Luciferase Reporter Gene Activity Assay

Since NFATc-1 served as downstream molecules of NF-κB and MAPK (Jiang et al., 2015), we employed NFATc-1 luciferase reporter gene assay on the basis of RAW264.7 cells that stably transfected with NFATc-1 luciferase reporter constructs, to determine the inhibitory effects of RbCl. Transfected RAW264.7 cells were treated with RbCl (1.6, 3.2 mM) and RANKL for 7 h, and luciferase activities were investigated using a Promega Luciferase Assay System (Madison, WI, USA) following the manufacturer’s instructions.

### Molecular Docking Assay

Using the architectures of human Jnk/p38 as templates, we constructed three-dimensional homology models of mouse Jnk/p38 kinase domains with Modeler 9.12. On the basis of AutoDock and AutoDock Vina (Trott and Olson, 2010), PROCHECK was used to verify the stereochemical structures of Jnk/p38, while Lamarckian genetic algorithm was utilized to link RbCl with kinases. The final figures of molecular docking indicating binding activity were presented with the PyMOL Visualization Software (Schrödinger LLC, New York, NY, USA).

### Alkaline Phosphatase (ALP) Staining and Quantification

Osteoblast MC3T3-E1 cells were seeded in 24-well plates at a density of 5.0 × 10^4^ cm^−2^ and treated with an osteogenic inductive medium including 100 nM dexamethasone, 50 μg/ml ascorbic acid, 10 mM β-glycerophosphate sodium, and different concentrations of RbCl (low: 1.6 mM; high: 3.2 mM). The medium was refreshed every 2 days until the 14th day. ALP staining was performed according to previously reported procedures (Tan et al., 2012), and ALP activity was determined using an ALP microplate test kit (Beyotime Bioscience, Shanghai, China), with the absorbance recorded at an optical density of 520 nm (OD_520_). The results were normalized to the corresponding total protein content using a BCA Protein Assay Kit (Thermo Scientific, IL, USA).

### Alizarin Red S (ARS) Staining and Quantification

Osteoblast MC3T3-E1 cells were seeded in 24-well plates at a density of 5.0 × 10^4^ cm^−2^ and treated with an osteogenic inductive medium including 100 nM dexamethasone, 50 μg/ml ascorbic acid, 10 mM β-glycerophosphate sodium, and different concentrations of RbCl (low: 1.6 mM; high: 3.2 mM). The medium was refreshed every 2 days until the 21st day. Cells were then fixed in 4% PFA for 15 min at 37°C before being stained with 1% ARS solution (Sigma-Aldrich) for 50 min. PBS was used to wash the non-specific staining, and representative images were taken by optical microscopy. For the quantitative assay, 10% cetylpyridinium chloride in 10 mM sodium phosphate was administered to stained cells, followed by the determination of optical density at 620 nm (OD_620_).

### Ovariectomized (OVX) Mouse Model

This study was carried out in accordance with the recommendations of guiding principles of Animal Care Committee of Central South University, and the protocol was approved by the Animal Care Committee of Central South University. Eight-week-old C57BL/6 mice (*n* = 24) were obtained from Shanghai Slac Laboratory Animal Company (Slac, Shanghai, China). Mice were kept in plastic-isolator cages under specific pathogen-free (SPF) conditions for 7 days to acclimatize, before OVX surgery was performed on them. Based on previously described procedures (Liu et al., 2017), mice were administered with intraperitoneal ketamine and xylazine mixture for anesthetization and analgesia. Next, a retroperitoneal incision was made ventral to the *erector* spinae muscles just caudal to the last rib to provide access to the ovary. The ovary was removed with catgut ligature located around the cranial portion of the uterus and uterine vessels, after which the incision was closed and mice were transported to warm conditions for recovery. After ovary resection, mice were randomly assigned into four groups: Sham (non-OVX mice), Vehicle (OVX mice), low (OVX mice injected with 4.5 mg/kg.bw RbCl intraperitoneally, three times/week), and high (OVX mice injected with 9 mg/kg.bw RbCl intraperitoneally, three times/week; *n* = 6 for each group; the doses of RbCl used were screened after preliminary selections *in vivo*). Sham and Vehicle mice received an injection of sterile PBS three times a week. Mice were injected and observed for 4 weeks.

### Titanium (Ti) Particle-Induced Calvarial Osteolysis Model

We also used a mouse calvarial osteolysis model to assess the effects of RbCl as described previously (Ouyang et al., 2014). Eight-week-old C57BL/6 mice (*n* = 24) were obtained from Shanghai Slac Laboratory Animal Company and kept in plastic-isolator cages under SPF condition for 7 days to acclimatize, before model establishment. Mice were administered with intraperitoneal ketamine for anesthetization, and the cranial periosteum was separated from calvarium by sharp dissection. Afterward, titanium particles (30 mg) were imbedded under the periosteum at the central suture of the calvaria. After Ti particle embedment, mice were randomly assigned into four groups and received drug administrations as mentioned above for the OVX model. Mice were injected and observed for 10 days.

### μCT Scanning

After treatment with RbCl, mice were euthanized, and the harvested tibiae and calvaria were fixed in 4% PFA before being transferred into a μCT scanning (μCT 40, Scanco, Zurich, Switzerland). The parameters used for the μCT tomography at a resolution of 10 μm were 80 kV (X-ray voltage) and 80 μA (electric current). Relevant bone volume fractions (BV/TV, %), trabecular number (Tb.N, 1/mm), trabecular separation (Tb.Sp, mm), and trabecular thickness (Tb.Th, mm) were obtained to analyze the effects of RbCl on OVX mice. The region of interest (ROI) in proximal tibia was defined to cover the whole subchondral bone in tibiae plateaus. Furthermore, the square ROI around the midline suture of the calvaria was selected, and relevant bone volume fractions (BV/TV, %), number of porosities, and the percentage of total porosities (%) were measured to evaluate the effects of RbCl on wear-particle induced osteolysis mice.

### Immunohistochemical Staining Analyses

After μCT analysis, harvested tibiae and calvaria were decalcified in 10% EDTA for 4 weeks, followed by paraffin-embedded procedures. Tissues were sectioned and stained for both hematoxylin-eosin (HE) and TRAP solution following the manufacturer’s guidelines.

### Statistical Calculations

All the data acquired from both *in vitro* and *in vivo* experiments were presented as means ± standard deviations (SD) and analyzed with SPSS 13.0 software (SPSS Inc., USA), using one-way analysis of variance. A two-tailed *p* < 0.05 was considered as statistically significant. All the experiments were repeated biologically at least three times independently.

## Results

### RbCl Attenuates Osteoclast Formation and Function *in vitro* Without Causing Cytotoxicity

**Figure 1:**
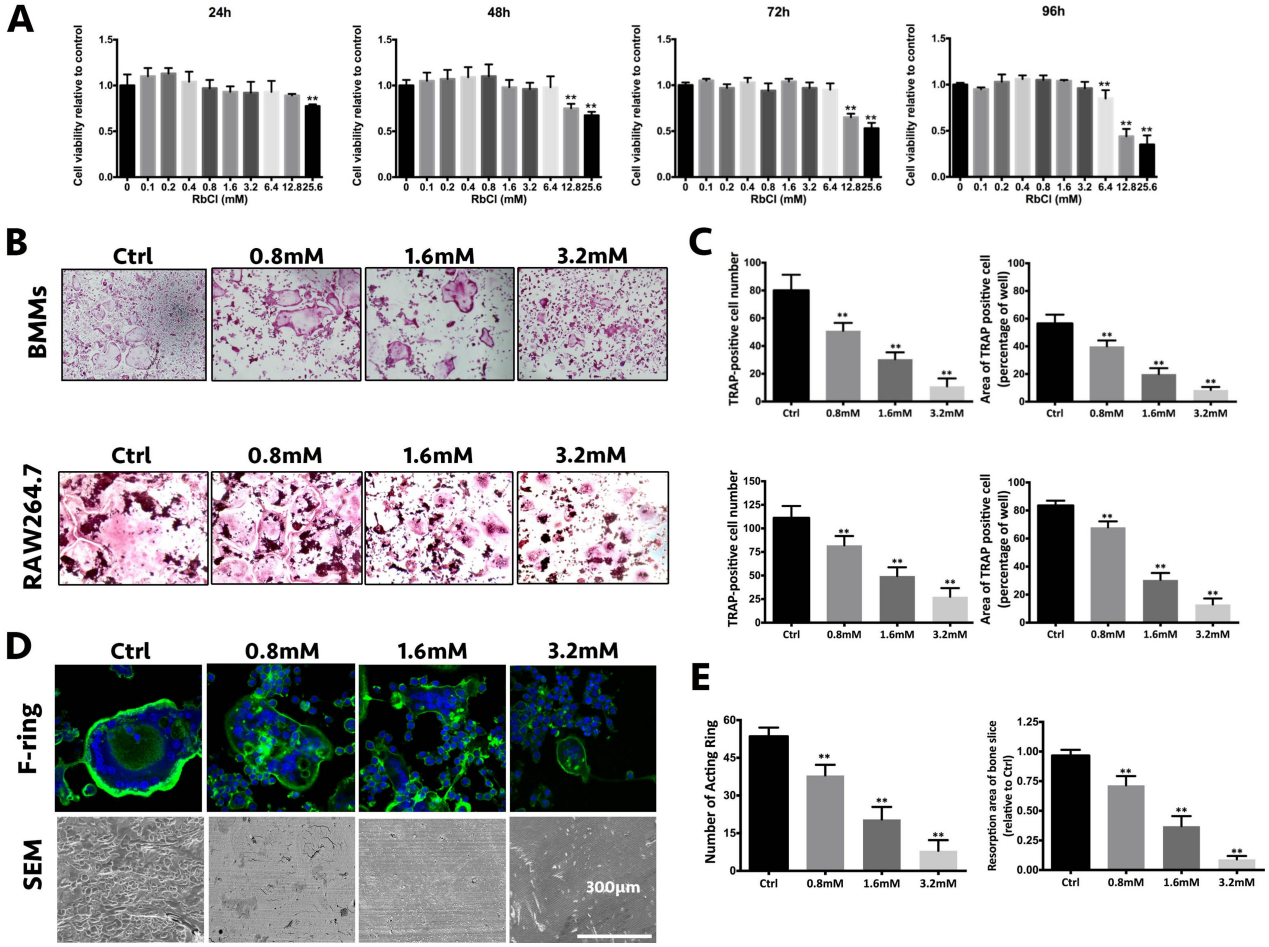
RbCl attenuates osteoclast formation and function without causing cytotoxicity *in vitro.* **(A)** Cell viability of osteoclast precursors after RbCl treatment from 24 to 96 h. **(B)** *In vitro* osteoclast formation of primary BMMs and RAW264.7 cells after RANKL and RbCl treatment. **(C)** Quantification of osteoclastogenesis by RbCl. **(D)** Formation of F-actin ring and bone resorption pits after RANKL and RbCl treatment. **(E)** Quantification of F-actin ring and bone resorption pits. *p < 0.05 compared with control. Each experiment was repeated biologically in triplicate independently.

To investigate the inhibitory effects of RbCl on osteoclast formation and function, we first sought to explore the non-toxic concentrations of RbCl upon osteoclast precursors and to exclude the direct suppressive effects upon osteoclast proliferation of RbCl. As shown in Figure 1A, only the highest concentration of RbCl (25.6 mM) showed significant osteoclast inhibitory effects after 24 h. With the increase of incubation time, it was evident that 25.6, 12.8, and 6.4 mM RbCl were able to exert significant osteoclast inhibition after 96 h, suggesting a dose-dependent action of RbCl on osteoclast proliferation. Therefore, non-toxic RbCl of 0.8, 1.6, and 3.2 mM were selected for further anti-osteoclastogenesis assessment. We found that for BMMs and RAW 264.7 cells, despite significant osteoclast formation showing large, round, and red-stained multinucleated osteoclasts in Ctrl, RbCl treatments decreased the number and area of the matured TRAP-positive osteoclasts dose-dependently (Figures 1B,C), signifying that RbCl could attenuate osteoclast formation significantly, without causing cytotoxicity.

Furthermore, since the formation of a well-shaped F-actin ring is indispensable for osteoclast functionality, the regulatory effects of RbCl on F-actin formation were also investigated. It was shown that in the control group, RANKL administration stimulated osteoclast to develop characteristically polarized F-actin ring. However, RbCl-mediated intervention led to a dramatic decrease in the F-actin rings, in terms of size and number (Figures 1D,E), implying that osteoclast functionality was damaged by the RbCl treatments. As expected, large and deep bone resorption pits were formed on bone slices of BMMs upon RANKL stimulation. Conversely, treatment with RbCl decreased both the number and the area of bone resorption pits on bone slices, showing the potential suppressive effects of RbCl on osteoclast formation and functionality *in vitro*.

### RbCl Impairs RANKL-Induced Osteoclast Gene Expression

**Figure 2:**
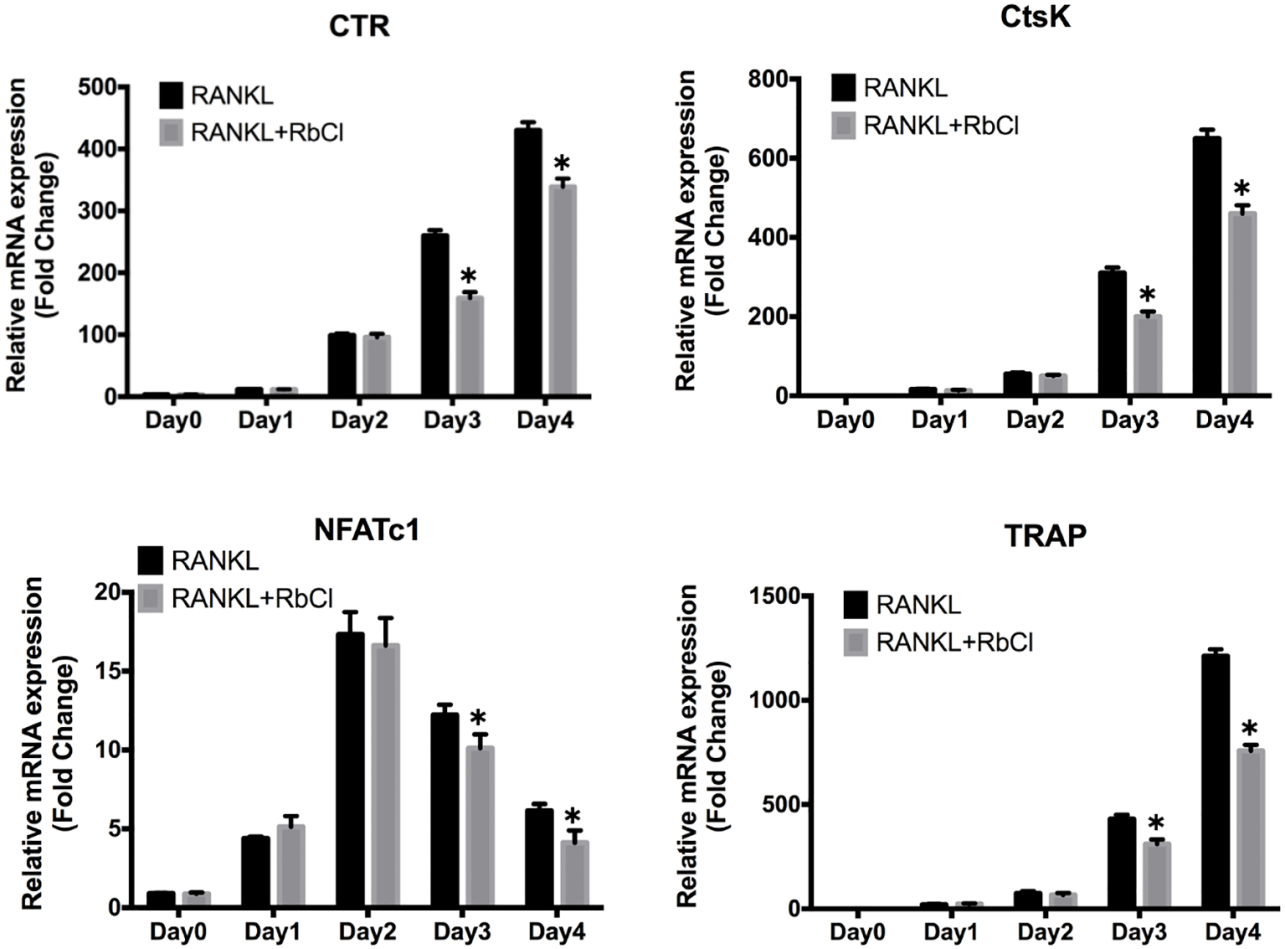
RbCl impairs RANKL-induced osteoclast gene expression. Evaluation of mRNA levels of osteoclast-specific genes in osteoclast precursors. *p < 0.05 compared with RANKL-treatment only. Each experiment was repeated biologically in triplicate independently.

During osteoclast formation, RANKL stimulates the expression of particular genes in osteoclasts, which lead to the differentiation from osteoclast precursors to matured osteoclasts (Boyle et al., 2003). As shown in Figure 2, it was evident that along with the progress of osteoclast differentiation, expression of osteoclastic markers CTR, CtsK, and TRAP increased steadily after RANKL stimulation. However, in comparison with RANKL-treatment only, the cells treated with RbCl and RANKL showed significantly decreased expression of CTR, CtsK, and TRAP. Furthermore, despite upregulation of NFATc-1 level in the first 2 days after RANKL administration, no statistical difference was seen after treatment with RbCl and RANKL. By contrast, the expression of NFATc-1 decreased during the following 2 days, indicating a significant inhibition of NFATc-1 by RbCl compared with the control. These results demonstrated that treatment with RbCl attenuated the production of osteoclast-specific genes during osteoclastogenesis *in vitro*.

### RbCl Suppresses RANKL-Induced Jnk/p38-Mediated NF-κB Co-activation

We aimed to unravel the effects of RbCl on RANKL-induced MAPK and NF-κB co-activation during osteoclast formation, thereby inhibiting the expression of osteoclast-specific genes.

**Figure 3:**
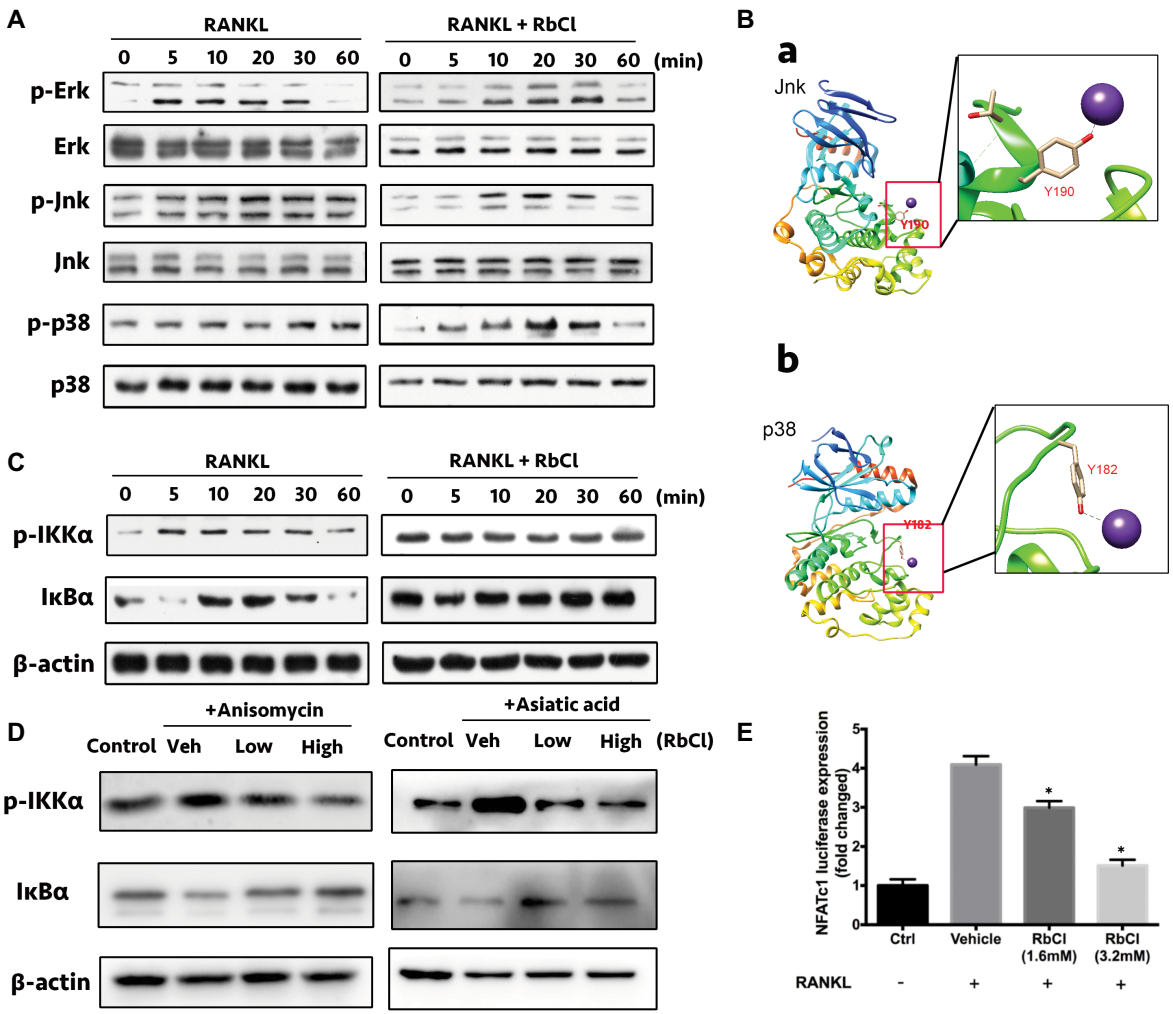
RbCl suppresses RANKL-induced Jnk/p38-mediated NF-κB co-activation. **(A)** Evaluation of suppressive effects on RANKL-stimulated MAPK signaling by RbCl. **(B)** Molecular docking of RbCl with Jnk/p38 kinases. **(C)** Evaluation of inhibitory effects on RANKL-induced NF-κB signaling by RbCl. **(D)** Transfected RAW264.7 cells were incubated with RbCl and RANKL, and luciferase activity for NFATc-1 was evaluated and normalized to the control. **(E)** Assessment of NF-κB activation after treatment with anisomycin (Jnk/MAPK activator), Asiatic acid (p38/MAPK activator), RANKL (50 ng/ml), and RbCl (low: 1.6 mM; high: 3.2 mM). *p < 0.05 compared with Vehicle. Each experiment was repeated biologically in triplicate independently.

As shown in Figure 3A, RANKL induced the increase of p-Erk at 5, 10, 20, and 30 min compared with that at 0 min, while RbCl delayed this tendency, as evidenced by the expression of p-Erk at 10, 20, and 30 min after RbCl treatment. Interestingly, we found that Jnk and p38 were steadily phosphorylated after RANKL stimulation, which was significantly attenuated after RbCl administration, indicating that RbCl could target phosphorylation of Jnk and p38, thereby inhibiting RANKL-stimulated MAPK activation.

Based on the above results, we attempted to determine the potential binding sites between RbCl with Jnk/p38 kinases by molecular docking analysis. As expected, results showed that RbCl could be embedded into the binding pockets of both Jnk and p38 kinases by establishing molecular bonds with tyrosine 190 and 182, respectively (Figure 3B). These results indicated that by targeting Jnk and p38 kinases, RbCl could possibly inhibit RANKL-induced MAPK activation, thereby attenuating osteoclast formation.

It was shown that constitutive activation of MAPK was associated with the increased phosphorylation of NF-κB signaling (Dhawan and Richmond, 2002; Korus et al., 2002; Papa et al., 2004), demonstrating the latent crosstalk between NF-κB and MAPK. Herein, we found that compared with 0 min of RANKL stimulation, the expression of p-IKKα increased within 30 min and decreased at 60 min. However, this tendency was invalidated by RbCl, which showed no significant differences 60 min after RANKL administration. Furthermore, IκBα was significantly degraded at 5 and 60 min, signifying the potent activity of IκBα after RANKL stimulation. Conversely, RbCl treatment inhibited the degradation of IκBα, resulting in the significant inactivation of the NF-κB pathway (Figure 3C).

Further, anisomycin (Jnk/MAPK activator) and Asiatic acid (p38/MAPK activator) were used to administer RANKL-stimulated osteoclasts to uncover the underlying connection between NF-κB and MAPK during osteoclastogenesis after RbCl treatments. We found that despite the activation of p-IKKα and IκBα after activator/RANKL administration, RbCl inhibited p-IKKα and increased IκBα expression significantly in osteoclasts (Figure 3D). Such results confirmed our hypothesis, that by targeting Jnk/p38-mediated NF-κB activation, RbCl tends to attenuate RANKL-induced osteoclastogenesis significantly, *in vitro*.

Next, since RbCl inactivated RANKL-induced MAPK and NF-κB, we aimed to unravel the alteration of downstream molecules (NFATc-1) of MAPK and NF-κB (Jiang et al., 2015). Herein, luciferase assay was deployed, showing that NFATc-1 was increased after RANKL administration. However, both low and high concentrations of RbCl tended to attenuate the activity of NFATc-1 dose-dependently (Figure 3E), signifying that RbCl inhibited the levels of NFATc-1 after targeting upstream NF-κB and MAPK during osteoclast formation.

### RbCl Promotes Osteoblastogenesis *in vitro*

**Figure 4:**
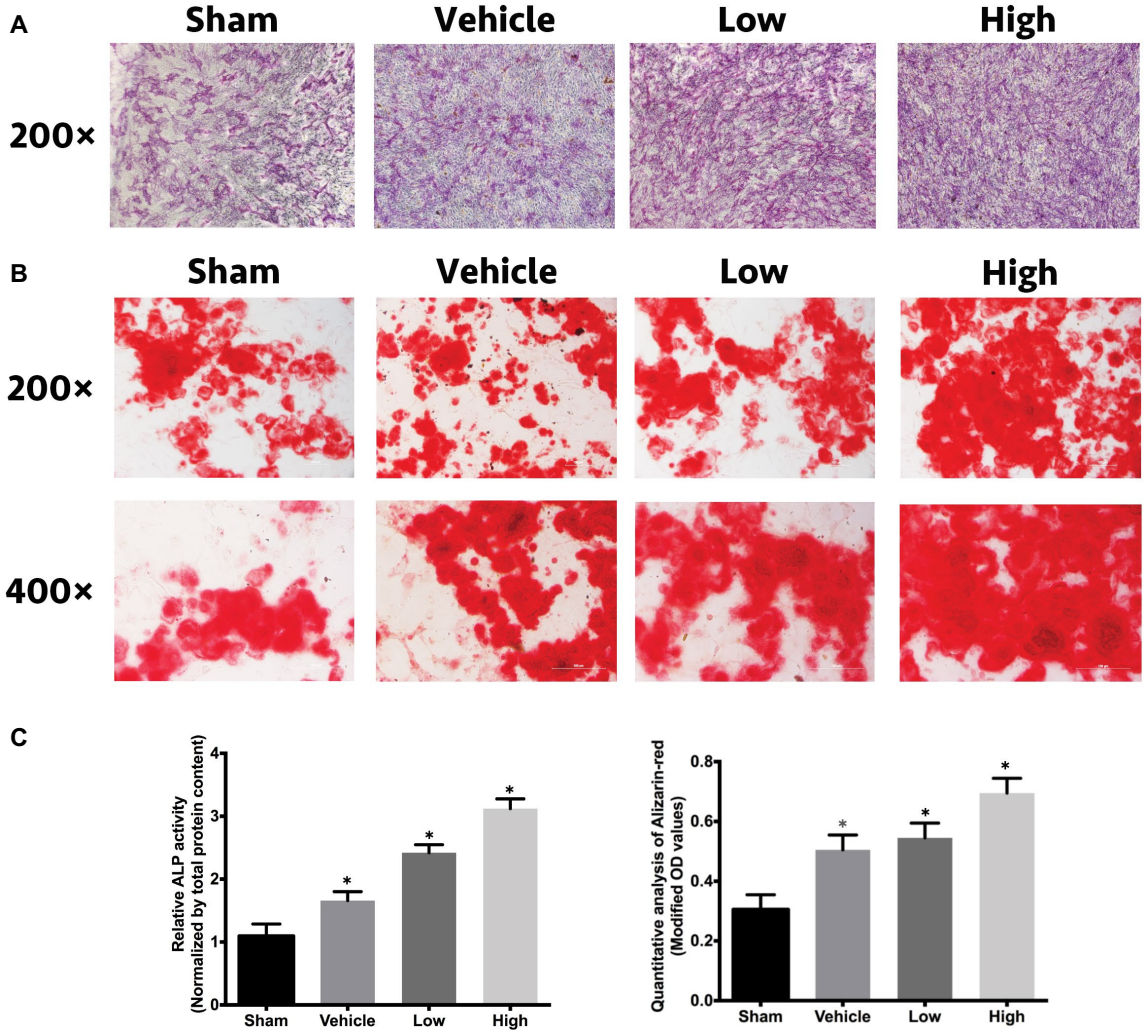
RbCl promotes osteoblastogenesis *in vitro.* Inductive effects of RbCl on osteogenesis of MC3T3-E1 cells. **(A)** ALP staining after 14 days of cell culture in osteogenesis medium with RbCl (magnification: 200×). **(B)** ARS staining after 21 days of cell culture in osteogenesis medium with RbCl (magnification: 200× and 400×) (low: 1.6 mM; high: 3.2 mM). **(C)** Quantitative analysis of ALP activity and ARS staining. *p < 0.05 compared with the Sham group. Each experiment was repeated biologically in triplicate independently.

ALP, ARS, and relative quantitative analysis were performed to comprehensively understand the effects of RbCl on osteoblastogenesis. Figures 4A–C showed that in comparison with Sham, the intensity of ALP and ARS in Vehicle was considerably stronger. Also, the intensities of osteoblastogenesis after RbCl treatment were even higher than those of Vehicle, signifying that RbCl could exhibit significant osteogenesis inductive effects despite its anti-osteoclastogenesis ability, which may provide a promising toolkit for the treatment of osteoporosis.

### RbCl Inhibits Bone Loss in OVX Mice

**Figure 5:**
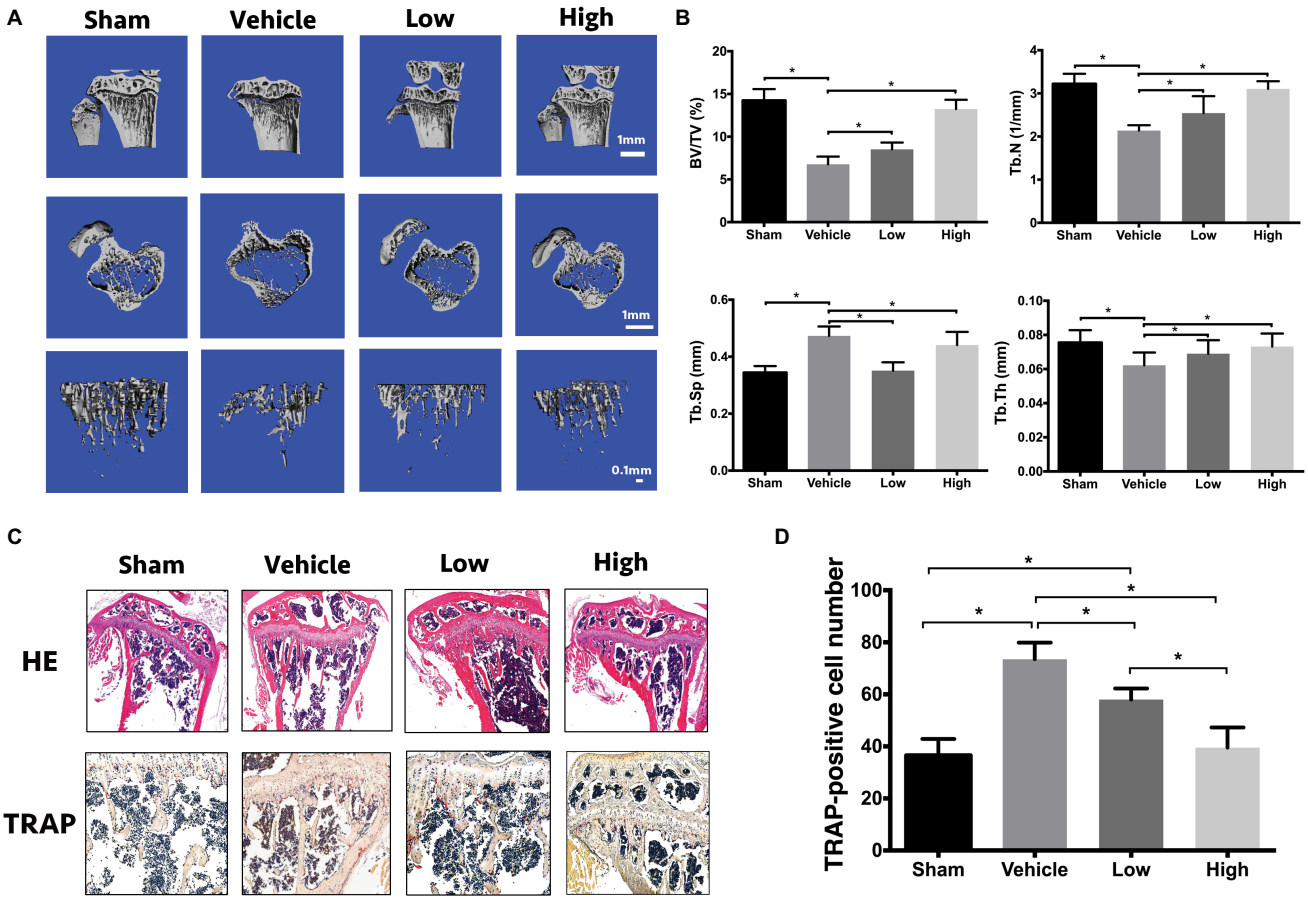
RbCl inhibits bone loss in OVX mice. **(A)** μCT tomography of tibiae of OVX mice. **(B)** Quantitative analyses from μCT scanning revealing BV/TV, Tb.N, Tb.Sp, and Tb.Th. **(C)** HE, TRAP staining of tibiae in OVX mice (magnification: 200×). **(D)** Quantitative analysis of TRAP-positive osteoclasts in tibiae of OVX mice. *p < 0.05. Each experiment was repeated biologically in triplicate independently.

After the evaluation of the effects of RbCl on osteoclasts and osteoblasts *in vitro*, the OVX mice model was employed to investigate the treatment effects of RbCl *in vivo*. As shown in Figure 5A, μCT showed an evident bone loss in Vehicle mice (OVX model). However, treatments of RbCl *in vivo* tended to attenuate such tendency, as exemplified by the better-preserved trabecular structure and bone density. Figure 5B of bone histomorphometric evaluation further demonstrated that compared with the Sham group, BV/TV, Tb.N, and Tb.Th decreased while Tb.Sp increased in the Vehicle group, showing osteoporosis phenotype *in vivo*. By contrast, the values of BV/TV, Tb.N, and Tb.Th increased, whereas Tb.Sp decreased after RbCl administration, implying that RbCl could inhibit bone loss in OVX mice.

Also, immunohistochemical analyses (Figure 5C) of HE staining showed the sparse trabecular architecture in tibiae in Vehicle in comparison with Sham, which was rescued by the administration of RbCl with intact knee joint and dense trabecular microarchitecture. Furthermore, increased intensity of TRAP-positive osteoclasts was observed in the Vehicle group, compared with that in Sham. However, RbCl downregulated the number of TRAP-positive osteoclasts (Figure 5D), showing the preservation of bone mass that resulted in the potential preservation of RbCl against bone loss *in vivo*.

### RbCl Suppresses Ti Particle-Induced Osteolysis *in vivo*

**Figure 6:**
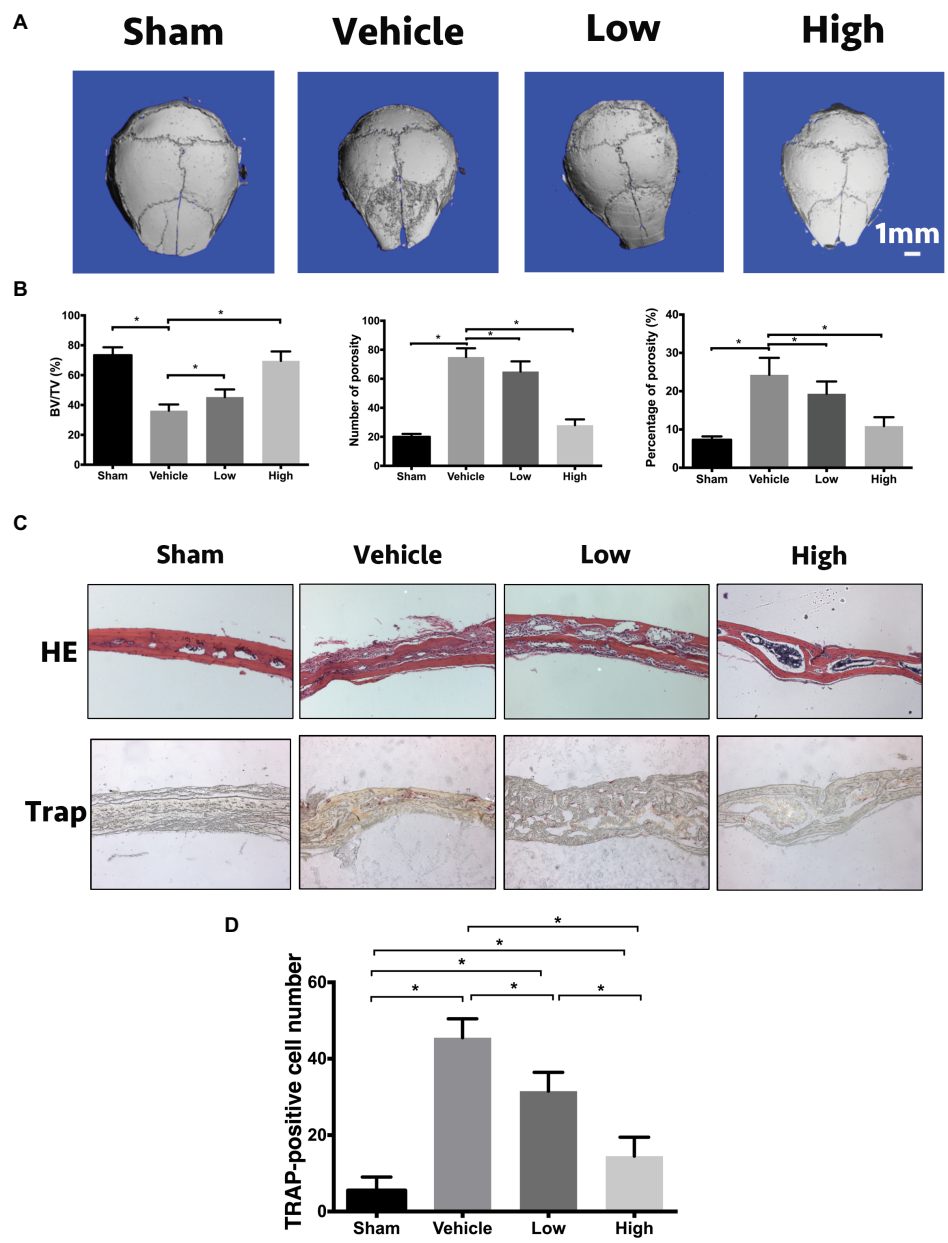
RbCl suppresses Ti particle-induced osteolysis *in vivo*. **(A)** μCT tomography of calvaria in Ti particle-induced osteolysis mice model. **(B)** Quantitative analyses from μCT scanning revealing BV/TV, number/percentage of porosity. **(C)** HE, TRAP of calvaria in Ti particle-induced osteolysis mice (magnification: 200×). **(D)** Quantitative analysis of TRAP-positive osteoclasts in calvaria in Ti particle-induced osteolysis mice. *p < 0.05. Each experiment was repeated biologically in triplicate independently.

In addition to OVX mice, we also used another osteolysis model to assess the effects of RbCl against osteoclastogenesis. As shown in Figure 6A, in comparison with Sham mice, a large area of osteolysis was observed in the Vehicle group. After RbCl treatment, Ti particle-induced bone resorption was inhibited considerably in a dose-dependent manner, implicating the significant suppressive effects of RbCl upon the formation of particle-induced osteolytic lesions. Besides, within the ROI area of calvaria, Ti particle induced significant decrease in BV/TV and increase in number/percentage of porosity (Figure 6B), which was abrogated by the treatments of RbCl in varying level compared with that in the Sham group, indicating the potential protective effects of RbCl against bone loss caused by Ti particle *in vivo*.

Furthermore, based on immunohistochemical analyses, we found that the administration of Ti particles induced the infiltration of lymphocytes and macrophages into calvaria significantly (Figure 6C). However, such inflammation was inhibited by the administration of RbCl, showing the alleviation of inflammatory infiltration and intact trabecular microstructure. Also, TRAP staining illustrated the alignment of mature multinucleated osteoclasts around eroded bone lesions in Vehicle. RbCl treatments reduced the area of osteolytic lesions significantly, with a diminished number of TRAP-positive osteoclasts in calvaria osteolysis (Figure 6D). Collectively, *in vivo* mice model showed that RbCl inhibited bone loss caused by hormone dysfunction and Ti particles significantly, indicating the strong possibility for future clinical translation of RbCl, such as in the surface coating of orthopedic implants.

## Discussion

**Figure 7:**
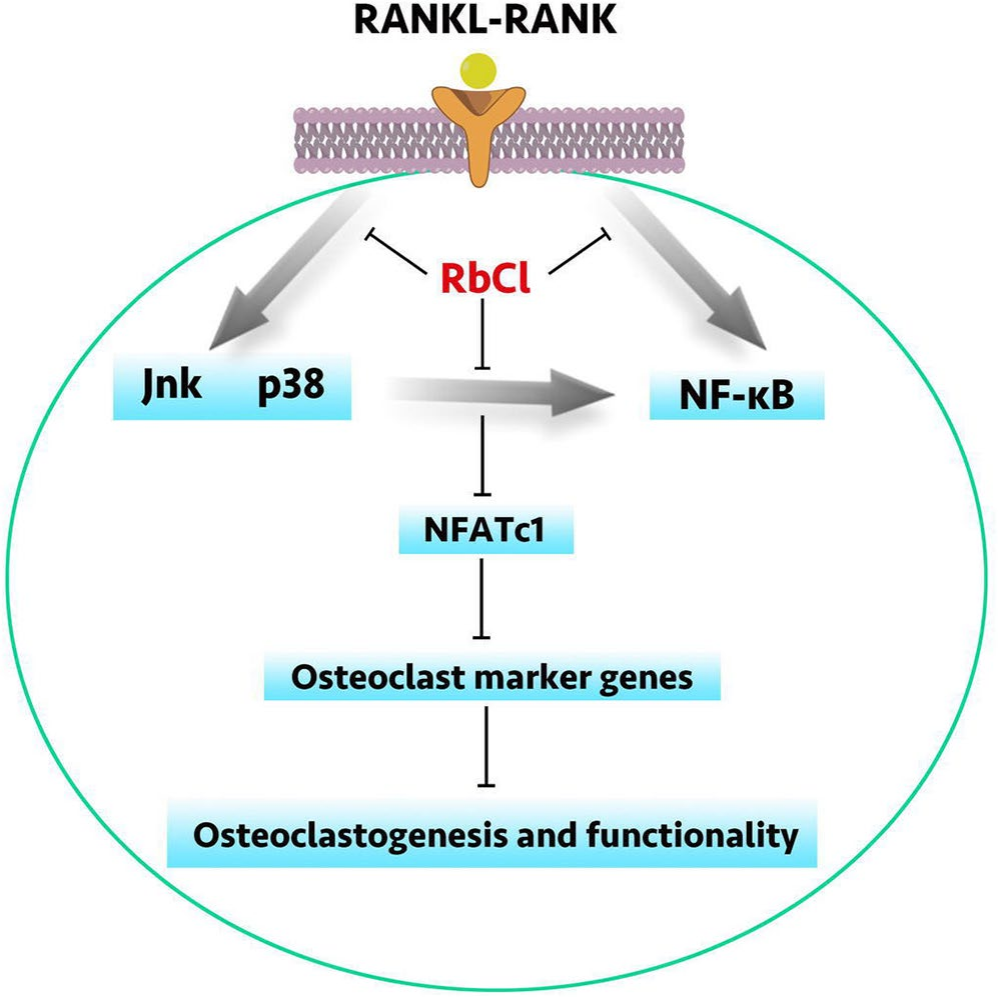
A schematic diagram of RbCl in regulating osteoclastogenesis and osteoblastogenesis. By targeting Jnk and p38-mediated NF-κB activation, RbCl attenuated RANKL-induced expressions of osteoclast marker genes to inhibit osteoclastogenesis and functionality *in vivo* and *in vitro*. Additionally, RbCl could also enhanced ALP activity and mineralization *in vivo* and *in vitro*, thereby re-establishing the homeostasis of bone microenvironment to provide the potent possibility for future translational practice in clinic, such as the surface coating of orthopedic implant.

The vivid crosstalk between MAPK and NF-κB has long been understood. However, current research seems insufficient to utilize this connection to inhibit osteoclast formation. Besides, in the condition of osteoporosis, it is of significance to attenuate osteoclastogenesis while facilitating osteoblastogenesis simultaneously. Herein, we have demonstrated that RbCl could inhibit RANKL-induced osteoclast formation and promote MC3T3-E1 cell osteoblastogenesis. Besides, by targeting Jnk/p38 molecules of MAPK signaling, RbCl not only inhibited the activation of RANKL-stimulated MAPK pathway but also ameliorated Jnk/p38-mediated NF-κB activation, thus providing potent inhibitory effects against osteoclastogenesis. (Figure 7) Also, on the basis of the two osteolysis animal models, we have shown the effective inhibition of RbCl against osteolysis induced by hormonal dysfunction and wear particles, extending the possible future utilization of RbCl into surface coating of orthopedic implants to protect against osteoporosis.

In the recent times, osteoporosis has become a serious issue worldwide, which necessitates effective medical and economic remedies to improve the life quality and longevity of afflicted patients. Mainly two approaches, antiresorptive and anabolic reagents, have been deployed to treat osteoporosis (Amugongo et al., 2014). Representative candidates for antiresorptive reagents included bisphosphonate and denosumab. Although desirable effects of anti-osteoclastogenesis were shown after treatment with antiresorptive reagents, patients could suffer from inevitable side effects (Qiao et al., 2016), which led to the exacerbation of illness significantly. Besides, serving as anabolic reagents, teriparatide (recombinant human parathyroid hormone) and romosozumab enhanced bone mineral density significantly (Neer et al., 2001; Ishibashi et al., 2017). Nonetheless, their efficacy and safety still further require in-depth Phase III study (Liu et al., 2017). Previously, RbCl has been used as an effective non-invasive soluble biomarker to assess the perfusion ability of myocardial cells (Hougardy et al., 2003). Also, it elevates the production of nitric oxide to exert antidepressant effects (Kordjazy et al., 2015). No literature is concerned about the application of RbCl in orthopedic translational approach, which highlights its potential as a novel coating biomaterial in bone implants in future.

Importantly, *in vitro* results showed that RbCl not only inhibited the osteoclast formation and functionality but also facilitated osteoblast differentiation significantly. To be more specific, RbCl attenuated RANKL-induced formation of TRAP-positive osteoclast, F-actin ring, and bone resorption pits, whereas it promoted the intensities of ALP and ARS of MC3T3-E1 cells simultaneously. These results highlighted the combination of antiresorptive and anabolic effects by RbCl, indicating a potent treatment strategy against osteoporosis.

As mentioned above, the process of osteoclastogenesis includes the activation of MAPK and NF-κB signaling. After the stimulation of RANKL, the extracellular stimulus was transferred into the nucleus for the upregulation of Jnk, p38, Erk, and NF-κB, resulting in the differentiation toward matured osteoclasts, with active expression of the osteoclast-specific genes CTR, Ctsk, NFATc-1, and TRAP. Herein, we found that RbCl was able to target NF-κB directly and indirectly. After the stimulation of Jnk/p38 activator and RANKL, RbCl inhibited the elevated expression of p-IKKα and the degradation of IκBα in osteoclast precursors, indicating indirect NF-κB inhibition *via* MAPK suppression. The resultant co-inactivation of MAPK and NF-κB by RbCl contributes to inhibit RANKL-stimulated osteoclast formation synergistically, as further evidenced by the downregulated levels of osteoclastic genes and activity of NFATc-1, highlighting that MAPK/NF-κB targeting strategy may serve as a promising approach in the treatment of osteoporosis.

Furthermore, deficiency of estrogen could result in the advent of osteoporosis. Besides, wear particle (Ti) derived from prosthesis used during total joint arthroplasty (TJA) could recruit and activate osteoclasts to local sites, leading to undesirable prosthetic loosening (Ouyang et al., 2014). Hence, we used both OVX and Ti particles-associated calvaria osteolysis mice models to evaluate the *in vivo* osteoclast inhibitory effects of RbCl extensively. Our results demonstrated that RbCl is beneficial for the protection of trabecular microarchitecture by inhibiting TRAP-positive osteoclastogenesis *in vivo*. Taken together, these findings indicated that RbCl inhibited RANKL-induced osteoclast formation both *in vitro* and *in vivo*. However, details on osteoblast activity *in vivo*, mechanisms regarding osteoblastogenesis, the weight changes of uterus, side effects of other physiologic tissues and organs after RbCl treatments should be taken into future consideration, which would facilitate the clinical translational practice of RbCl against osteoporosis. Also, the molecular docking between RbCl and p38/Jnk was largely speculative, which required further biochemical analyses in-depth.

In conclusion, we found a brand-new application of RbCl in medical translation practice, i.e., suppression of osteoclastogenesis while facilitating osteoblastogenesis simultaneously *via* Jnk/p38-mediated NF-κB activation, both *in vitro* and *in vivo*. Our finding suggests the potential possibility of using RbCl in the future for surface coating of orthopedic biomaterial implants to protect against osteoporosis.

## Ethics Statement

This study was carried out in accordance with the recommendations of guiding principles of Animal Care Committee of Central South University, and the protocol was approved by the Animal Care Committee of Central South University.

## Author Contributions

ZO carried out the molecular genetic studies, participated in the sequence alignment, and drafted the manuscript. QH and BL carried out the animal study. HW carried out the immunoassays and participated in the sequence alignment. YL participated in the design of the study and performed the statistical analysis. TL conceived of the study and participated in its design and coordination and helped to draft the manuscript. All authors read and approved the final manuscript.

## Conflict of Interest Statement

The authors declare that the research was conducted in the absence of any commercial or financial relationships that could be construed as a potential conflict of interest.

## Correction note

A correction has been made to this article. Details can be found at: 10.3389/fphar.2025.1678247.
