# Correction: Rubidium chloride targets Jnk/p38-mediated NF-κB activation to attenuate osteoclastogenesis and facilitate osteoblastogenesis

**DOI:** 10.3389/fphar.2025.1678247

**Published:** 2026-01-08

**Authors:** Zhengxiao Ouyang, Qianli Huang, Bin Liu, Hong Wu, Tang Liu, Yong Liu

**Affiliations:** 1 State Key Laboratory of Powder Metallurgy, Central South University, Changsha, China; 2 Department of Orthopedics, The Second Xiangya Hospital, Central South University, Changsha, China

**Keywords:** rubidium chloride, osteoclast, osteoblast, MAPK, NF-κB, osteoporosis

There was a mistake in Figure 1B as published. Specifically, the image for the control in the BMMs group was incorrectly selected during figure preparation. The affected image has been replaced with the correct representative image from the original experiments.

**FIGURE 1:**
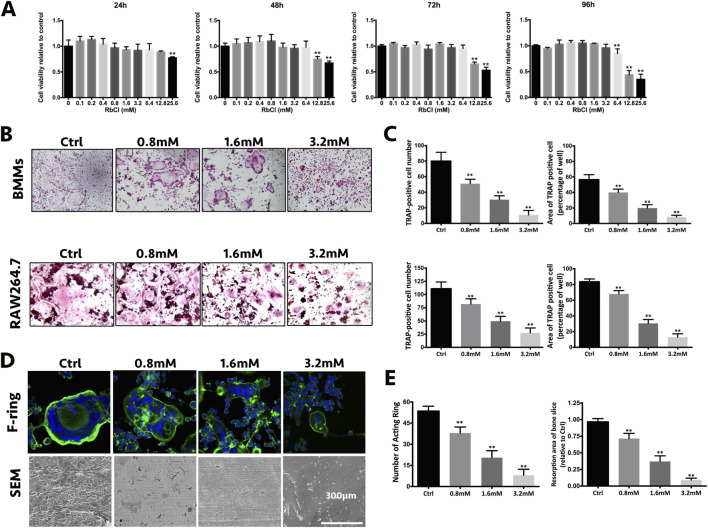
RbCl attenuates osteoclast formation and function without causing cytotoxicity *in vitro*. **(A)** Cell viability of osteoclast precursors after RbCl treatment from 24 to 96 h. **(B)** *In vitro* osteoclast formation of primary BMMs and RAW264.7 cells after RANKL and RbCl treatment. **(C)** Quantification of osteoclastogenesis by RbCl. **(D)** Formation of F-actin ring and bone resorption pits after RANKL and RbCl treatment. **(E)** Quantification of F-actin ring and bone resorption pits. *p < 0.05 compared with control. Each experiment was repeated biologically in triplicate independently.

The corrected Figure 1B appears below.

These changes do not affect the scientific conclusions of the article in any way.

The original article has been updated.

